# Attention-guided jaw bone lesion diagnosis in panoramic radiography using minimal labeling effort

**DOI:** 10.1038/s41598-024-55677-3

**Published:** 2024-02-29

**Authors:** Minseon Gwak, Jong Pil Yun, Ji Yun Lee, Sang-Sun Han, PooGyeon Park, Chena Lee

**Affiliations:** 1 Department of Electrical Engineering, Pohang University of Science and Technology, Pohang, 37673 Republic of Korea; 2 Manufacturing AI Research Center, Korea Institute of Industrial Technology, Incheon, 21999 Republic of Korea; 3 Department of Oral and Maxillofacial Radiology, Yonsei University College of Dentistry, Seoul, 03722 Republic of Korea; 4 Institute for Innovation in Digital Healthcare, Yonsei University, Seoul, 03722 Republic of Korea; 5 KITECH School, University of Science and Technology, Daejeon, 34113 Republic of Korea

**Keywords:** Dental diseases, Image processing, Dental diseases, Image processing

## Abstract

Developing a deep-learning-based diagnostic model demands extensive labor for medical image labeling. Attempts to reduce the labor often lead to incomplete or inaccurate labeling, limiting the diagnostic performance of models. This paper (i) constructs an attention-guiding framework that enhances the diagnostic performance of jaw bone pathology by utilizing attention information with partially labeled data; (ii) introduces an additional loss to minimize the discrepancy between network attention and its label; (iii) introduces a trapezoid augmentation method to maximize the utility of minimally labeled data. The dataset includes 716 panoramic radiograph data for jaw bone lesions and normal cases collected and labeled by two radiologists from January 2019 to February 2021. Experiments show that guiding network attention with even 5% of attention-labeled data can enhance the diagnostic accuracy for pathology from 92.41 to 96.57%. Furthermore, ablation studies reveal that the proposed augmentation methods outperform prior preprocessing and augmentation combinations, achieving an accuracy of 99.17%. The results affirm the capability of the proposed framework in fine-grained diagnosis using minimally labeled data, offering a practical solution to the challenges of medical image analysis.

## Introduction

Panoramic radiography, a fundamental imaging tool in dentistry, is typically the first line of defense when screening for jaw bone lesions^1^. Frequently encountered pathologies of the jaw bones are cysts, tumors, and tumor-like diseases. Extensive efforts have been made to develop deep-learning-based models for pathological diagnosis because identifying lesions on panoramic radiography is of significant clinical importance^1–7^. Despite these advances, there remains a noticeable gap in the availability of practical platforms that can be effectively used for diagnosing jaw bone pathologies under clinical conditions.

Research on deep learning-based pathological diagnosis can be broadly categorized based on the complexity of the tasks it aims to solve. A simple approach to this task involves training an image classification model. However, image-classification models primarily focus on categorizing the entire input image, which may limit their ability to classify specific regions within the entire image. Owing to this limitation, previous research implemented a system that a specific region of interest within the entire radiograph, a crop of the image, is classified into distinct diseases^4^. A more advanced approach involves object detection, which combines lesion detection with classification, thereby performing a higher-level task^5,6^. However, this approach necessitates the construction of diagnostic models where explicit model parameters are responsible for localization^8,9^. These models are built upon the robust assumption that the dataset is annotated with location labels. For example, Ariji et al.^5^ and Kwon et al.^6^ utilized 210 and 946 box-labeled training data, respectively, for the auto-diagnosis of jaw lesions.

Creating location labels is often complicated by the ambiguity in annotation criteria, which vary among dentists. Accurate labeling is particularly challenging owing to inconsistencies that arise from individual dentists and even among seasoned oral and maxillofacial radiologists. This challenge stems from the properties of panoramic radiography, which result in non-standardized images with severe superimposition and distortion of the dentomaxillofacial anatomy^10,11^. The ambiguity in defining annotation areas can be mitigated to some extent by recent studies that have proposed methods for training deep learning models amidst label inaccuracies^12–15^. However, generating location labels for all patient data remains an inevitably resource-intensive task, requiring considerable effort from dentists. Particularly for panoramic radiography depicting jaw lesions, it is posited that constructing a practical model would greatly benefit from the availability of highly precise labeled data. “Good quality data”, which have been strictly reviewed and agreed upon by multiple skilled radiologists, are essential for developing a clinically feasible diagnostic system. Considering the aforementioned challenges, this paper proposes a method that minimizes the labor required for labeling in diagnosing pathological conditions using dental panoramic images.

In this paper, a pathology diagnosis framework that leverages an advanced classification model was proposed to produce diagnostic predictions with enhanced class activation map (CAM)-based localization results, utilizing the least number of location labels from panoramic radiographs. In the proposed framework, attention guidance was employed by evaluating the intersection over union (IoU) between the attention map generated through gradient-weighted Class Activation Mapping (Grad-CAM)^16^ and the corresponding annotation provided by the radiologist. In the training process, an attention-guided feature extractor was designed to automatically extract both diagnostic and positional information from panoramic radiographs. In addition, multiple augmentation techniques were applied specifically tailored for panoramic radiographs to maximize the utility of the minimally labeled data. Notably, the novel trapezoid augmentation was applied, which randomly alters the ratio between the maxilla and mandible, leading to the utilization of data from virtual functional patients. The PyTorch implementation for the framework is available at https://github.com/msgwak/att-radiology.

The primary contributions of this work are summarized as follows:

(i) An attention-guided feature extractor was designed to automatically derive diagnostic and positional information from panoramic radiographs, leveraging Grad-CAM for binarized attention maps and measuring their scale-invariant alignments with corresponding labels. The effectiveness of the proposed framework was validated by consistent experiment results.
(ii) We demonstrated that even a few attention labels significantly enhance maxillofacial pathology diagnosis, with generated attention maps accurately highlighting lesion areas, thus aiding dentists in interpreting results.
(iii) Tailored data augmentation techniques, including trapezoid transformation, were introduced for panoramic radiographs. They can effectively expand the dataset and maximize the utility of minimally-labeled data, as evidenced by an ablation study.

## Review of relevant literature

### Automated diagnosis for jaw bone pathology

Many deep-learning-based diagnostic studies on panoramic radiography have been published, and the number is increasing rapidly. Among these, automatic diagnoses of jawbone pathology have been attempted in several studies. Most developed detection model for the relatively common and important diseases of the jaw, including ameloblastoma, odontogenic keratocyst, dentigerous cyst, and periapical cyst^4–6,17^. Lee et al.^4^ and Ariji et al.^5^ performed studies on cysts and tumor-mimicking lesions and investigated whether they could be distinguished from the major diseases using deep learning. Sensitivity to classify lesions was 0.98 in Lee et al.’s study with small sample sizes (*n*= 463)^4^. Ariji et al.^5^ reported classification sensitivity of these diseases was 0.13–0.82 and detection sensitivity was 0.71–1.00 using DetectNet^8^. The model performance was better in the study by Kwon et al.^6^, which utilized YOLOv3^9^ with a 1.5 times larger sample size; sensitivity ranged from 0.54 to 0.98, depending on the disease. One of the differences in the methods of these previous studies^4–6^ is the data annotation style. All previous studies created box-shaped labels for deep learning model training, whereas Lee et al. introduced an innovative approach of lesion border-specific annotations^4^. Generating these lesion border-specific annotations is a challenging process that requires considerable effort and time to amass large-scale high-quality data for model training. The proposed framework addresses this challenge by leveraging the available partially location-labeled data for network training, thereby alleviating this labeling burden.

### Object localization using attention maps

The development of a CAM in the field of deep learning has shown that a well-trained convolutional neural network (CNN) is capable of image localization. This discovery has sparked research on CAM-based object localization, in which meaningful features are captured within the input data without the need for explicit trainable parameters for localization that are commonly observed in traditional object detection models^8,9^. Ongoing investigations in the computer vision domain have often focused on refining activation maps in terms of the discriminative region or speed^18^. Moreover, the CAM results were downstream of the main task, for example, segmentation. Diba et al.^19^ utilized the CAM results obtained in the earlier stage as pseudo-labels for a segmentation task in the subsequent stage. Li et al.^20^ employed additional supervision and self-supervision to refine attention maps for use as priors in segmentation tasks.

Notably, CAM-based object localization has been successfully applied in various domains of medical imaging, including the diagnosis of brain lesions^21,22^, analysis of chest CT images^23^, and retinal fundus image analysis^24^. Despite these advancements, the utilization of such techniques in panoramic-radiograph-based diagnoses remains underexplored in the context of integration of object localization into medical imaging. We posit that the primary objective in diagnosing jawbone lesions from panoramic radiographs is simply to identify the location of the pathological regions, rather than requiring a detailed pixel-by-pixel segmentation of the entire image. Thus, by focusing on the localization of lesions and drawing inspiration from previous research^20^, this study investigates how a classification model, guided by a limited amount of supervision for attention maps, can achieve high performance with minimal labeling costs in the diagnosis of jaw bone conditions.

## Materials and methods

### Ethics

This study was approved by the institutional review board (IRB No. 2-2020-0084) of Yonsei University Dental Hospital and was conducted and completed in accordance with the ethical regulations. Due to the retrospective nature of the study, the requirement for informed consent was waived, and this was approved by Yonsei University Dental Hospital, IRB. All imaging data were anonymized before export.

**Table 1:** Number of samples by data type.

Type	Cyst and tumor			Tumor-like lesion	Normal
	Dentigerous cyst	Odontogenic keratocyst	Ameloblastoma	Lingual mandibular bone depression	No pathology
Amount	91	92	90	173	270
Subtotal	273			173	270
Total	716

**Table 2:** Number of training samples according to different proportions of attention labels.

Class	Label type	0%	5%	10%	20%	50%	100%
Normal	Class	170					
Cyst and Tumor	Class	172					
	Attention	0	14	23	44	86	172
LMBD	Class	87					
	Attention	0	2	6	9	40	87

The percentage indicates the proportion of attention labels.

### Data acquisition

Panoramic radiographs taken at our institution, showing commonly occurring lesions in jaw bone—including dentigerous cysts (DC), odontogenic keratocysts (OKC), and ameloblastomas (AB)—as confirmed by histopathology, were collected between January 2019 and February 2021. A tumor-like lesion, the lingual mandibular bone depression (LMBD), was confirmed using computed tomography. Normal cases without jaw bone pathologies were also collected. The total number of data points was 716, and the specific sample sizes for each class type are listed in Table 1. All radiographs were obtained using the following equipment: Rayscan Alpha Plus (Ray Co. Ltd., Hwaseong-si, Korea) with exposure conditions of 71 kvP, 12 mA, and 14.1 s; and Pax-i Plus (Vatech Co., Hwaseong-si, Korea) with exposure conditions of 71 kVp, 14 mA and 13.5 s. This study was approved by the Institutional Review Board (IRB) of Yonsei University Dental Hospital, and the requirement for informed consent was waived because of the retrospective nature of the study (IRB no. 2-2020-0084).

### Data labeling

**Figure 1:**
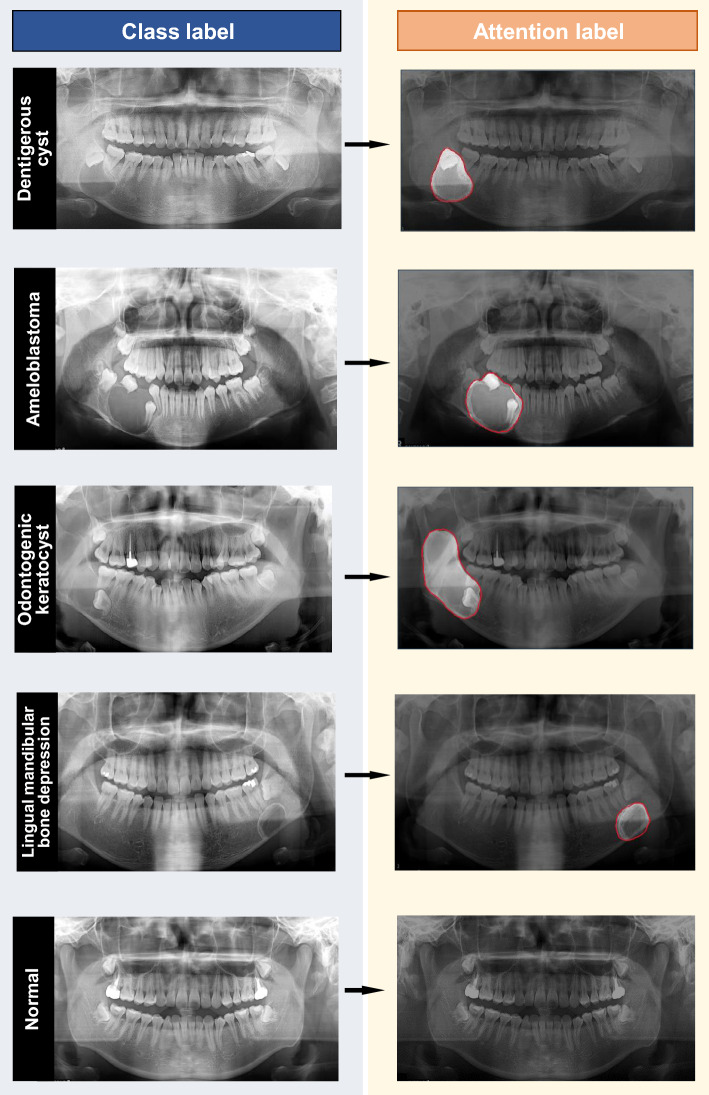
Data class label and attention label using box and border-specific annotation.

Two oral and maxillofacial radiologists performed the labeling process, and the region-of-interest (ROI) was determined on the borderline or periphery of each individual lesion using a free-form line (Fig. 1). To simulate scenarios in which attention labels were not available for the entire dataset, the usage of attention labels were controlled in our experiments. From the data selected for training, random subsets comprising 0%, 5%, 10%, 20%, 50%, and 100% were chosen to utilize attention labels for the lesion data, whereas the remainder were restricted from using attention labels. A proportion of 0% signifies the use of class labels without attention labels, whereas 100% indicates that attention labeling was performed for all data. Class labels were consistently used for all data, irrespective of the presence of attention labels. The actual number of classes and attention labels used in training, based on the proportion of attention labels utilized, is detailed in Table 2.

### Data augmentation

**Figure 2:**
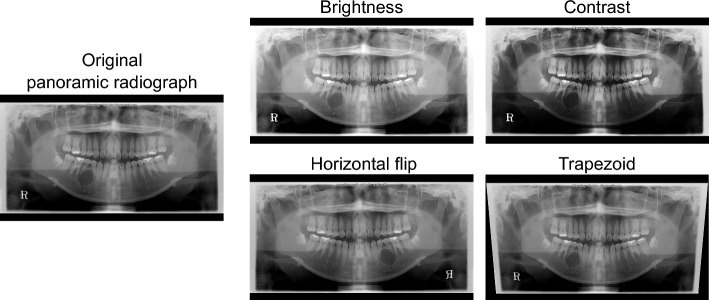
Data augmentation methods.

To expand the collected dataset virtually, data augmentation methods were applied on both the panoramic radiographs and attention labels. The augmentation process includes transformations simultaneously applied to both panoramic radiographs and corresponding attention labels, as well as transformations applied exclusively to panoramic radiographs. Figure 2 illustrates the methods used in this process. The brightness of the radiographs was randomly adjusted within a range of − 5% to + 5%, and the contrast was randomly altered within a range of − 10% to + 10%. This ensured variations in the intensity levels of the images. Additionally, the images were flipped horizontally with a 50% probability to enhance variability further. This operation mirrored the radiographs and induced variations in orientation. Furthermore, a trapezoid-shaping projective transformation, referred to as a trapezoid transformation, was applied to diversify the ratio of both jaws. Applying the trapezoid transformation, the base width of the radiographs was randomly increased or decreased within a range of − 5% to + 5%. Both horizontal flip and trapezoid transformations were applied equally to the panoramic radiographs and their corresponding attention labels. This ensures spatial alignment between the radiographs and labels, thereby preserving accurate and consistent annotations for the network attention.

### Data preprocessing

Subsequently, we applied a border-cropping operation to all the panoramic radiographs and attention labels. This process ensures that the model input is confined to the jaw bone region while excluding any unnecessary regions from the diagnostic process. Furthermore, the text marks indicating the institution and equipment model names were removed from the images. As a result, the border-cropping operation effectively prevented overfitting of the model. The sizes of the center cropping area were appropriately chosen based on the dimensions of the data used. Moreover, the operation was applied consistently during the inference phase to ensure consistent and reliable results.

### Attention-guided diagnosis framework

**Figure 3:**
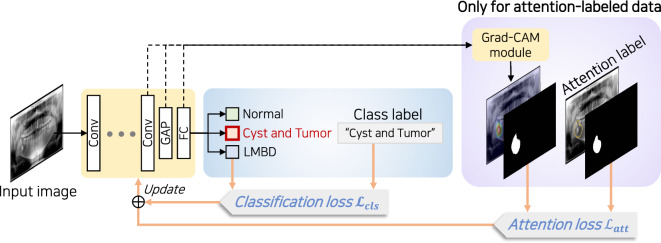
Framework overview.

The attention-guided diagnosis framework conditionally utilizes the attention map and its soft mask, contingent on the availability of attention labels for the data. Figure 3 illustrates the framework used in this study. For the backbone CNN-based classification model, *f*, we used a pretrained ResNet50 model^25^. The convolutional blocks in the backbone model encode an input image *x* into feature maps that capture the discriminative features present in the image. The feature maps are converted into a vector using a global average pooling (GAP) layer and then classified into specified classes with a fully connected (FC) layer.

In the training process of the proposed framework, feature maps of attention-labeled data are additionally passed through a Grad-CAM module^16^ to obtain the attention map $\textit{H}$, which highlights the influential region in the input image for the target classes. We leverage feature maps from the last convolutional layer, which captures high-level information from the input image. The Grad-CAM module first computes the gradient of the target class score with respect to feature maps. The importance weight of each feature map was then computed by applying GAP to the corresponding gradient. An attention map is then obtained by computing the weighted sum of the feature maps using importance weights. An attention label represents a binary mask, clearly indicating which regions within the input image contain a lesion, with values of either 0 or 1. In contrast, the attention map generated by Grad-CAM is distributed across the entire image. Therefore, attention maps need to be transformed into a form comparable to the labels while ensuring that differentiability is maintained throughout the process. To achieve this, we amplified values above a certain threshold $\theta \in [0,1]$, while diminishing values below, emphasizing the core regions of activation. The attention map $H\in {\mathbb {R}}^{m\times n}$ initially undergoes min-max normalization, resulting in ${\bar{H}}\in [0,1]^{m\times n}$ where values lie between 0 and 1. Subsequently, a soft mask ${\tilde{H}}\in \{0,1\}^{m\times n}$ is obtained using a sigmoid function as follows:

1 $$\begin{aligned} {\tilde{H}} = s(\omega ({\bar{H}}-\theta J_{m\times n})), \end{aligned}$$

where $\omega$ is a scaling parameter, typically set to a large number for thresholding, e.g., 100, $J_{m\times n}$ is an *m*-by-*n* all-ones matrix, and $s(\cdot )$ is defined as

2 $$\begin{aligned} s(z) = \frac{1}{1+e^{-z}}. \end{aligned}$$

### Optimization objective

The optimization objective of the training classifier $\textit{f}$ is twofold: enhancing the classification performance and inducing attention to pertinent regions. Under the assumption that every data point is annotated by a class label, the classification loss is computed across all data. By contrast, attention loss is calculated exclusively for data annotated with attention labels.

For classification loss, we employed the cross-entropy metric to distinguish between the normal and lesion samples. For image *x* and its class label *y*, the formulation of the classification loss is as follows:

3 $$\begin{aligned} {\mathscr {L}}_{cls} = -\log \frac{e^{f_y(x)}}{\sum _{k=1}^{N_c} e^{f_k(x)}}, \end{aligned}$$

where $f_k(x)$ denotes the logit value for class *k* when data *x* is input into a model *f* and $N_c$ is the total number of classes.

In addition, we employ an attention loss to enhance the differentiation between disease and disease-like lesions. If there exists an attention label *Y* for a sample *x*, we obtain an attention map and convert it into a soft mask ${\tilde{H}}$ according to (1). The soft mask is then compared to the attention label using the IoU metric. The computation for the attention loss is detailed as follows:

4 $$\begin{aligned} {\mathscr {L}}_{att} = {\left\{ \begin{array}{ll} 1-\text {IoU}({\tilde{H}}, Y), &{} \quad \text {if } Y \text { exists}\\ 0, &{} \quad \text {otherwise} \end{array}\right. }, \end{aligned}$$

where

5 $$\begin{aligned} \text {IoU}(A,B) = \frac{\text {Area of}~ A\cap B}{\text {Area of}~ A\cup B}, \end{aligned}$$

for $A, B\in \{0,1\}^{m\times n}$. The IoU metric effectively mitigates the influence of varying lesion sizes on the magnitude of the loss, ensuring a more consistent and unbiased optimization process.

By integrating (3) and (4), the final optimization objective ${\mathscr {L}}$ can be expressed as follows:

6 $$\begin{aligned} {\mathscr {L}} = \alpha _{cls} {\mathscr {L}}_{cls} + \alpha _{att} {\mathscr {L}}_{att}, \end{aligned}$$

where $\alpha _{cls}$ and $\alpha _{att}$ denote weights for ${\mathscr {L}}_{cls}$ and ${\mathscr {L}}_{att}$, respectively.

## Experiment

### Experimental setup

The experimental setup for the model training was as follows. As computational resources, we used an AMD EPYC 7513 32-Core Processor (AMD, Santa Clara, California, USA) and an NVIDIA RTX A6000 (Santa Clara, California, USA). The attention label usage rate was adjusted proactively, starting with no usage (rate:0) and gradually increasing to full usage (rate:1) in increments of 0.05, 0.1, 0.2, and 0.5 intervals. This method allowed us to observe the effect of attention label usage on the diagnostic performance. The data were distributed across the training, validation, and testing sets in a ratio of 6:2:2, respectively. For data augmentation, we applied intensity augmentation with a brightness factor of 0.05 and a contrast factor of 0.1. We also incorporated a horizontal flip augmentation with a probability of 0.5 and a jaw ratio augmentation with a factor of 0.05. Following data augmentation, we performed a center cropping operation on the panoramic radiographs, which originally had dimensions of $1280\times 720 \,\hbox {pixels}$, and yielded images with dimensions of $940 \times 520 \,\hbox {pixels}$.

We used a ResNet50 feature extractor pre-trained with ImageNet^26^ and an FC layer for classification was initialized by Kaiming uniform initialization^27^. The FC layer was trained using a strategy of dropout^28^ with a rate of 0.25 to mitigate overfitting. The model was optimized by a stochastic gradient descent scheme with an initial learning rate of $10^{-3}$ and a weight decay of $10^{-5}$. Cosine annealing learning rate scheduling^29^ was utilized, setting the maximum number of iterations, $T_{max}$, to 50. The model was trained for 100 epochs for a batch size of 8. Two loss weights, $\alpha _c$ and $\alpha _{att}$, were set to 2 and 1, respectively. The final model was selected based on its highest validation accuracy with an early stopping mechanism for a patience level of 40 epochs to prevent overfitting.

### Evaluation metric

The accuracy, sensitivity, and specificity of each trained model were assessed. The metrics were first calculated for each class and macro-averaged, treating the class in the evaluation as positive and all other classes as negative. The accuracy, defined in Eq. (7), quantifies the proportion of correct predictions (both positive and negative) out of the total number of test samples.

7 $$\begin{aligned} \text {Accuracy}=\frac{\text {TP}+\text {TN}}{\text {TP}+\text {TN}+\text {FP}+\text {FN}}, \end{aligned}$$

where TP, TN, FP, and FN denote the number of true positives, true negatives, false positives, and false negatives, respectively. Sensitivity, as defined in Eq.  (8), measures the proportion of correctly identified positives out of the total number of actual positives.

8 $$\begin{aligned} \text {Sensitivity}=\frac{\text {TP}}{\text {TP}+\text {FN}}. \end{aligned}$$

Specificity, defined as in (9), measures the proportion of correctly identified negatives out of the total number of actual negatives.

9 $$\begin{aligned} \text {Specificity}=\frac{\text {TN}}{\text {TN}+\text {FP}}. \end{aligned}$$

Moreover, all reported results were computed through five repeated experiments.

### Result

**Figure 4:**
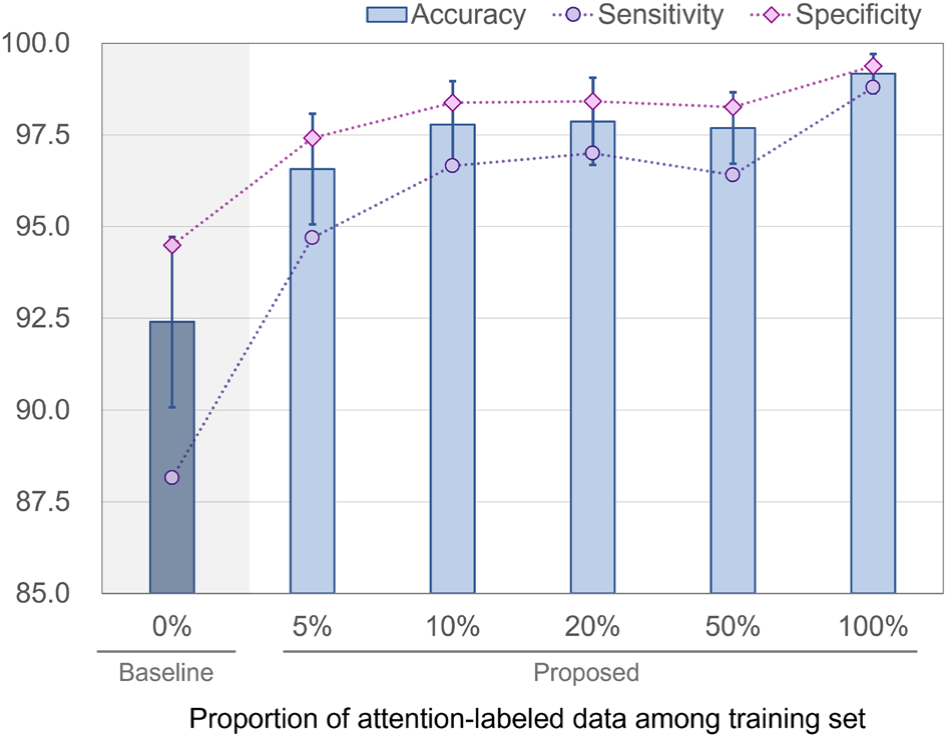
Plot of metric evaluation results for different attention label usage rates in training.

**Figure 5:**
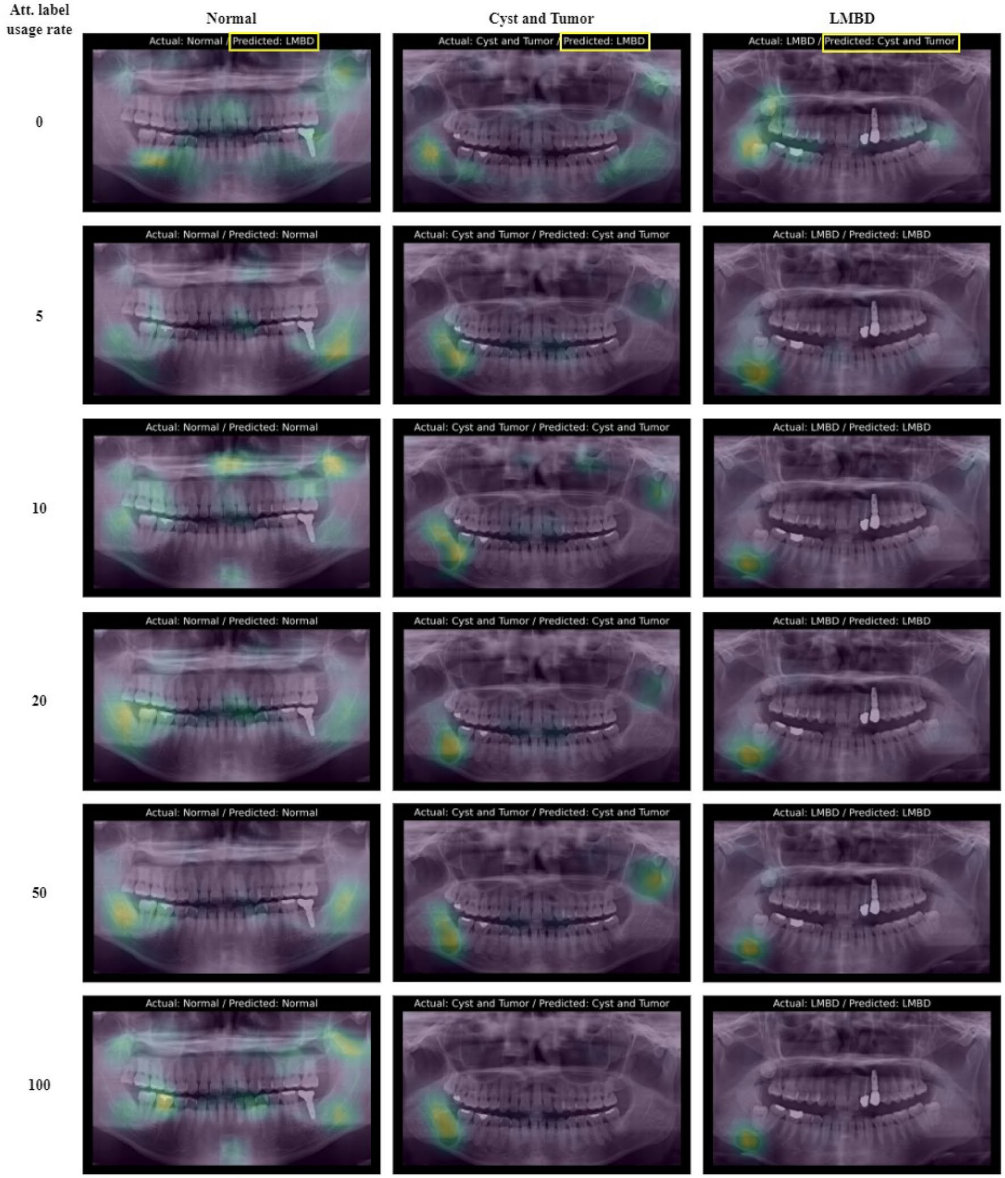
Diagnosis results with network attention visualization.

The evaluation results for the models trained with varying attention-label usage rates are presented in Fig. 4. The exact values of the figure and detailed results can be seen in Table 3. The models achieved accuracies of 92.41% and 99.17%, when trained with 0% and 100% attention-labeled data, respectively. As the proportion of attention-labeled training data increased, the model performance demonstrably increased in terms of accuracy, sensitivity, and specificity. This suggests that an increase in the proportion of attention-labeled training data potentially enhances the model’s predictive capabilities. Notably, even with a mere 5% attention-label usage rate, the accuracy was 96.57%. An interpretation of the significant performance improvement, even with minimal attention labels, can be provided through the attention map visualizations shown in Fig. 5.

**Table 3:** Metric evaluation results for different attention label usage rates in training.

Metric	0% (Baseline)	5%	10%	20%	50%	100%
Accuracy	$92.41 \pm 2.32$	$96.57 \pm 1.51$	$97.78 \pm 1.03$	$97.87 \pm 1.19$	$97.69 \pm 0.97$	$99.17 \pm 0.54$
Sensitivity	$88.16 \pm 3.51$	$94.69 \pm 2.44$	$96.66 \pm 1.55$	$97.00 \pm 1.71$	$96.41 \pm 1.64$	$98.80 \pm 0.80$
Specificity	$94.49 \pm 1.68$	$97.42 \pm 1.17$	$98.38 \pm 0.76$	$98.42 \pm 0.87$	$98.26 \pm 0.74$	$99.38 \pm 0.41$

The percentage indicates the proportion of attention labels.

Figure 5 displays the ground truth, predicted class, and attention map for the same panoramic-radiograph cases differentiated by the proportion of attention labels. Specifically, it highlights cases in which the predictions from a classification model trained solely with class labels differ from the ground truth. In the normal class, the attention of the models was distributed across both jaws. For the cysts, tumors, and LMBD classes, models trained only with class labels often misdirect their attention toward other regions, such as the teeth, rather than the actual lesion. By contrast, the proposed model trained with attention labels focuses more accurately on the lesion, leading to more precise class predictions.

**Figure 6:**
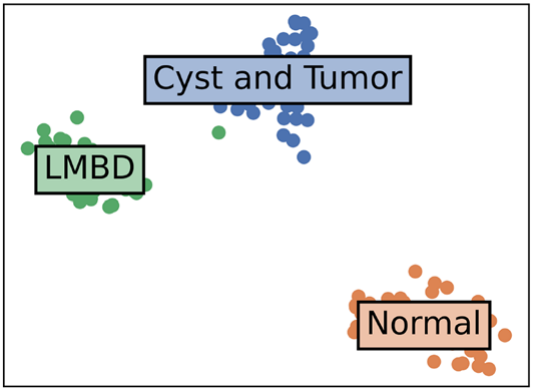
T-SNE visualization of learned features. Left: proportion of attention labels 0%. Right: proportion of attention label 100%.

Furthermore, Fig. 6 shows the GAP-processed feature vectors for test samples in 2D space using t-distributed stochastic neighbor embedding (t-SNE)^30^ for models trained with attention label proportions of 0% and 100%. For 0% proportion, the features of the Cyst and Tumor class and the LMBD class data were clustered with ambiguous boundaries. However, at a 100% proportion, the features in the two classes formed distinct clusters, as evident from the results in (). The model trained with attention labels extracted features that were more conducive to differentiation than the model trained solely with class labels. This implies that guiding the network attention toward pertinent lesion areas through partial supervision can effectively distinguish between challenging lesions.

### Ablation study on data augmentation methods

The influence of data augmentation methods was investigated, particularly those tailored for panoramic radiographs. Initially, we constructed the set of comparison methods used in a previous study^6^. This set encompasses an image-processing method known as contrast-limited adaptive histogram equalization (CLAHE)^31^. CLAHE preprocesses the contrast of the input images using patchwise computations. Furthermore, the set comprised three data augmentation methods: intensity, flip, and rotation. Applying the hyperparameter settings from this previous study^6^, we configured the rotation augmentation method to randomly rotate an input image within a range of − 1 to 1 deg. Subsequently, various combinations of image processing and data augmentation methods were explored by adjusting different hyperparameters, as presented in Table 4. Based on our exploration, the optimal preprocessing settings include intensity, flip, and trapezoid augmentation. The results show that the trapezoid augmentation alone improves performance. Moreover, when used in conjunction with other effective augmentation combinations, it results in a notable increase in accuracy. Based on empirical observations, e omitted combinations that included CLAHE or rotation augmentation from the result table, as CLAHE might be redundant for intensity augmentation, and rotate augmentation tends to be more effective for other datasets specifically containing a high prevalence of twisted jaw cases. For reference, although the comparison method achieved an accuracy of 95.69%, the proposed method reached an average of 99.17% across five independent experiments. From these results, it is evident that every added method contributes to a performance enhancement. This improvement can be attributed to the capability of these methods to expand patient datasets, which suffer from data collection challenges.

**Table 4:** Effectiveness of data augmentation methods.

Image processing	Augmentation	Accuracy
CLAHE	Intensity + Flip + Rotate	$95.69 \pm 0.92$
–	–	$94.50 \pm 1.27$
–	Intensity	$96.25 \pm 2.18$
–	Flip	$97.50 \pm 0.99$
–	Trapezoid	$96.11 \pm 2.22$
–	Intensity + flip	$97.50 \pm 0.83$
–	Intensity + trapezoid	$95.74 \pm 2.47$
–	Flip + trapezoid	$97.50 \pm 1.59$
–	Intensity + flip + trapezoid	${{\textbf {99.17}}} \pm {{\textbf {0.54}}}$

Significant values are in bold.

## Discussion

### Clinical convenience: a comparison with conventional image classification and object detection methods

Our methodology offers a clinically viable solution for the diagnostic process using deep learning models. Traditional image classification models require cropping of the lesion region for both training and inference when aiming for fine-grained classification. In contrast, the proposed methodology directly diagnoses raw panoramic radiographs, eliminates the cropping process, and streamlines the diagnostic procedure. Although object detection methods can employ raw panoramic radiographs, they require location-specific labels for all data. Our proposed approach minimizes the necessary labeling, particularly in scenarios with limited labeled data. Through experiments, we validated that our method significantly enhances the diagnostic performance, even with a small amount of labeled data. Practically, it is difficult to create a single, accurate label that is reviewed by multiple experts. In most studies, a single radiologist or non-specialist is trained to label^3,5^. Considering the complex nature of medical imaging, determining the exact region of pathology requires a high degree of effort, which means that the labeling data used in many studies may contain imperfectly curated ones. This study attempted to solve such realistic limitations in deep learning research based on medical images. It can be expected that further application of the suggested model in the current study into other types of diagnostic imaging would benefit from minimum data curation labor with enhanced accuracy.

### Fine-grained classification for medical imaging

In our training methodology, for samples with attention labels, an additional attention loss was computed to refine the network attention. Unlike conventional L2 loss, our proposed method utilizes IoU loss, thereby mitigating the influence of lesion area size. By enhancing the network attention, our model focused on lesion areas with high clinical relevance within the entire radiographs, thereby avoiding overfitting to nonlesioned areas in the training images. Consequently, the model showed the capability of fine-grained classification to distinguish between cysts, tumors, and LMBD in experiments without the need for extensive labeling.

### Crafting data augmentation for panoramic radiography

To devise a data-augmentation method specifically tailored for panoramic radiographs, we rigorously assessed general techniques, discarding less beneficial ones while introducing novel approaches to expand a given dataset better. The data augmentation techniques in medical imaging can be restricted in that any method producing the diagnostic imaging that cannot exist in actual clinical conditions should not be simulated. For example, a vertical flip (up-side-down) of the image cannot exist in actuality, specifically in panoramic radiography. Likewise, the data augmentation method in panoramic radiography is restricted to histogram adjustment. However, changing the histogram of the radiography is often unsuitable for model training. Moreover, hue adjustment, a popular choice in image classification research, is deemed inappropriate when grayscale input images are used for training and testing. Similarly, the CLAHE image preprocessing method can improve image clarity; however, it has occasionally been observed to excessively brighten some images, raising concerns about its reliability and the possibility of producing images that interfere with learning. Rotation augmentation was conducted within a subtle rotation range to preserve data consistency, which yielded negligible performance improvement in our experiments. As such, data augmentation is the inevitable procedure for medical imaging training study. However, the method is very limited. Thus, this study established a new augmentation method specific to panoramic radiography, the so-called trapezoid method. This type of image augmentation can occur in real-world clinical settings. Panoramic radiographs are characterized by frequent horizontal magnification of the maxillofacial bone. Therefore, the trapezoid method is thought to be a suitable augmentation technique. Based on these observations, our methodology integrated flipping, intensity, and novel trapezoid augmentation. Trapezoid augmentation compensated for the limitation of data acquisition owing to the diverse jaw proportions encountered across patients, leading to a more generalizable and accurate diagnostic model.

### Limitation

In this study, although the proposed framework reduces the constraints related to location labels compared to traditional object detection methods, it still requires a small number of location labels and induces direct supervision for attention, which is a limitation. Ideally, to achieve an advanced diagnosis without relying on location labels, it would be beneficial to employ methods that guide the attention of the model through self-guidance. Therefore, there is a need for research on self-guidance methods tailored to the unique characteristics of panoramic radiographs.

## Conclusion

In this study, we introduced a framework capable of enhancing the diagnosis of maxillofacial pathologies with minimal labeling effort. Our approach leverages available attention labels and incorporates them into a conditionally computed additional loss. Through rigorous analysis, we identified an optimal combination of data augmentation methods tailored for panoramic radiograph data to address the challenges associated with the limited availability of medical imaging data. Notably, the augmentations were applied to all the data, maximizing the utility of the available attention-labeled data. Experimental evaluations of our dataset demonstrate the effectiveness of our framework. Remarkably, even when labeling was restricted to only 5% of the data, we observed significant improvements in both the accuracy and visualization of model attention.

Finally, future work will improve the model using attention information. One promising research direction is to leverage self-attention guidance for a more fine-grained classification of cysts and tumors. We believe that research employing attention information will lead to the development of generalizable models based on limited data.

## Data Availability

The data generated and analyzed during the current study are not publicly available due to privacy laws and policies in Korea, but are available from the corresponding author on reasonable request.

## References

[1] Choi J-W (2011). Assessment of panoramic radiography as a national oral examination tool: Review of the literature. Imaging Sci. Dent..

[2] Song I-S (2022). Deep learning-based apical lesion segmentation from panoramic radiographs. Imaging Sci. Dent..

[3] Lee J-H (2020). Application of a fully deep convolutional neural network to the automation of tooth segmentation on panoramic radiographs. Oral Surg. Oral Med. Oral Pathol. Oral Radiol..

[4] Lee A (2021). Deep learning neural networks to differentiate Stafne’s bone cavity from pathological radiolucent lesions of the mandible in heterogeneous panoramic radiography. PLoS ONE.

[5] Ariji Y (2019). Automatic detection and classification of radiolucent lesions in the mandible on panoramic radiographs using a deep learning object detection technique. Oral Surg. Oral Med. Oral Pathol. Oral Radiol..

[6] Kwon O (2020). Automatic diagnosis for cysts and tumors of both jaws on panoramic radiographs using a deep convolution neural network. Dentomaxillofac. Radiol..

[7] MacDonald D, Yu W (2020). Incidental findings in a consecutive series of digital panoramic radiographs. Imaging Sci. Dent..

[8] Tao A (2016). Detectnet: Deep neural network for object detection in digits. Parallel Forall.

[9] Redmon, J. & Farhadi, A. Yolov3: An incremental improvement. Preprint at http://arxiv.org/abs/1804.02767 (2018).

[10] Kim H-S (2023). Refinement of image quality in panoramic radiography using a generative adversarial network. Dentomaxillofac. Radiol..

[11] Mallya S, Lam E (2019). White and Pharoah’s Oral Radiology E-book: Principles and Interpretation: Second South Asia Edition E-Book.

[12] Gudivada V (2017). Data quality considerations for big data and machine learning: Going beyond data cleaning and transformations. Int. J. Adv. Softw..

[13] Song H (2022). Learning from noisy labels with deep neural networks: A survey. IEEE Trans. Neural Netw. Learn. Syst..

[14] Reed, S. *et al.* Training deep neural networks on noisy labels with bootstrapping. Preprint at http://arxiv.org/abs/1412.6596 (2014).

[15] Simpson, A. L. *et al.* A large annotated medical image dataset for the development and evaluation of segmentation algorithms (2019).

[16] Selvaraju, R. R. *et al.* Grad-cam: Visual explanations from deep networks via gradient-based localization. In *Proc. IEEE Int. Conf. Comput. Vis.* 618–626 (2017).

[17] Kuwana R (2021). Performance of deep learning object detection technology in the detection and diagnosis of maxillary sinus lesions on panoramic radiographs. Dentomaxillofac. Radiol..

[18] Shao, F. *et al.* Deep learning for weakly-supervised object detection and object localization: A survey. Preprint at http://arxiv.org/abs/2105.12694 (2021).

[19] Diba, A. *et al.* Weakly supervised cascaded convolutional networks. In *Proc. IEEE Comput. Vis. Pattern Recognit.* 914–922 (2017).

[20] Li, K. *et al.* Tell me where to look: Guided attention inference network. In *Proc. IEEE Comput. Soc. Conf. Comput. Vis. Pattern Recognit.* 9215–9223 (2018).

[21] Dubost F (2020). Weakly supervised object detection with 2d and 3d regression neural networks. Med. Image Anal..

[22] Ji, Z. *et al.* Scribble-based hierarchical weakly supervised learning for brain tumor segmentation. In *Medical Image Computing and Computer Assisted Intervention—MICCAI 2019: 22nd International Conference, Shenzhen, China, October 13–17, 2019, Proceedings, Part III 22* 175–183 (Springer, 2019).

[23] Wang X (2020). A weakly-supervised framework for covid-19 classification and lesion localization from chest ct. IEEE Trans. Med..

[24] Gondal, W. M. *et al.* Weakly-supervised localization of diabetic retinopathy lesions in retinal fundus images. In *2017 IEEE International Conference on Image Processing (ICIP)* 2069–2073 (IEEE, 2017).

[25] Simonyan, K. & Zisserman, A. Very deep convolutional networks for large-scale image recognition. Preprint at http://arxiv.org/abs/1409.1556 (2014).

[26] Deng, J. *et al.* Imagenet: A large-scale hierarchical image database. In *Proc. IEEE Comput. Vis. Pattern Recognit.* 248–255 (IEEE, 2009).

[27] He, K. *et al.* Delving deep into rectifiers: Surpassing human-level performance on imagenet classification. In *Proc. IEEE Int. Conf. Comput. Vis.* 1026–1034 (2015).

[28] Srivastava N (2014). Dropout: A simple way to prevent neural networks from overfitting. J. Mach. Learn. Res..

[29] Loshchilov, I. & Hutter, F. Sgdr: Stochastic gradient descent with warm restarts. Preprint at http://arxiv.org/abs/1608.03983 (2016).

[30] Van der Maaten L, Hinton G (2008). Visualizing data using t-sne. J. Mach. Learn. Res..

[31] Reza AM (2004). Realization of the contrast limited adaptive histogram equalization (CLAHE) for real-time image enhancement. J. VLSI Sig. Proc. Syst..

